# Nanomechanics of tip-link cadherins

**DOI:** 10.1038/s41598-019-49518-x

**Published:** 2019-09-16

**Authors:** Javier Oroz, Albert Galera-Prat, Rubén Hervás, Alejandro Valbuena, Débora Fernández-Bravo, Mariano Carrión-Vázquez

**Affiliations:** 1 Instituto Cajal/CSIC, Avda. Doctor Arce, 37, E-28002 Madrid, Spain; 2 Present Address: Instituto de Química Física Rocasolano/CSIC, C/Serrano 119, E-28006 Madrid, Spain; 3 Present Address: Stowers Institute for Medical Research, 1000 East 50th Street, Kansas City, MO 64110 USA; 4 Present Address: Centro de Biología Molecular “Severo Ochoa” (CSIC-Universidad Autónoma de Madrid), Universidad Autónoma de Madrid, Cantoblanco, E-28049 Madrid, Spain

**Keywords:** Single-molecule biophysics, Cadherins

## Abstract

Hearing and balance rely on the transduction of mechanical stimuli arising from sound waves or head movements into electrochemical signals. This archetypal mechanoelectrical transduction process occurs in the hair-cell stereocilia of the inner ear, which experience continuous oscillations driven by undulations in the endolymph in which they are immersed. The filamentous structures called tip links, formed by an intertwined thread composed of an heterotypic complex of cadherin 23 and protocadherin 15 ectodomain dimers, connect each stereocilium to the tip of the lower sterocilium, and must maintain their integrity against continuous stimulatory deflections. By using single molecule force spectroscopy, here we demonstrate that in contrast to the case of classical cadherins, tip-link cadherins are mechanoresilient structures even at the exceptionally low Ca^2+^ concentration of the endolymph. We also show that the D101G deafness point mutation in cadherin 23, which affects a Ca^2+^ coordination site, exhibits an altered mechanical phenotype at the physiological Ca^2+^ concentration. Our results show a remarkable case of functional adaptation of a protein’s nanomechanics to extremely low Ca^2+^ concentrations and pave the way to a full understanding of the mechanotransduction mechanism mediated by auditory cadherins.

## Introduction

**Figure 1:**
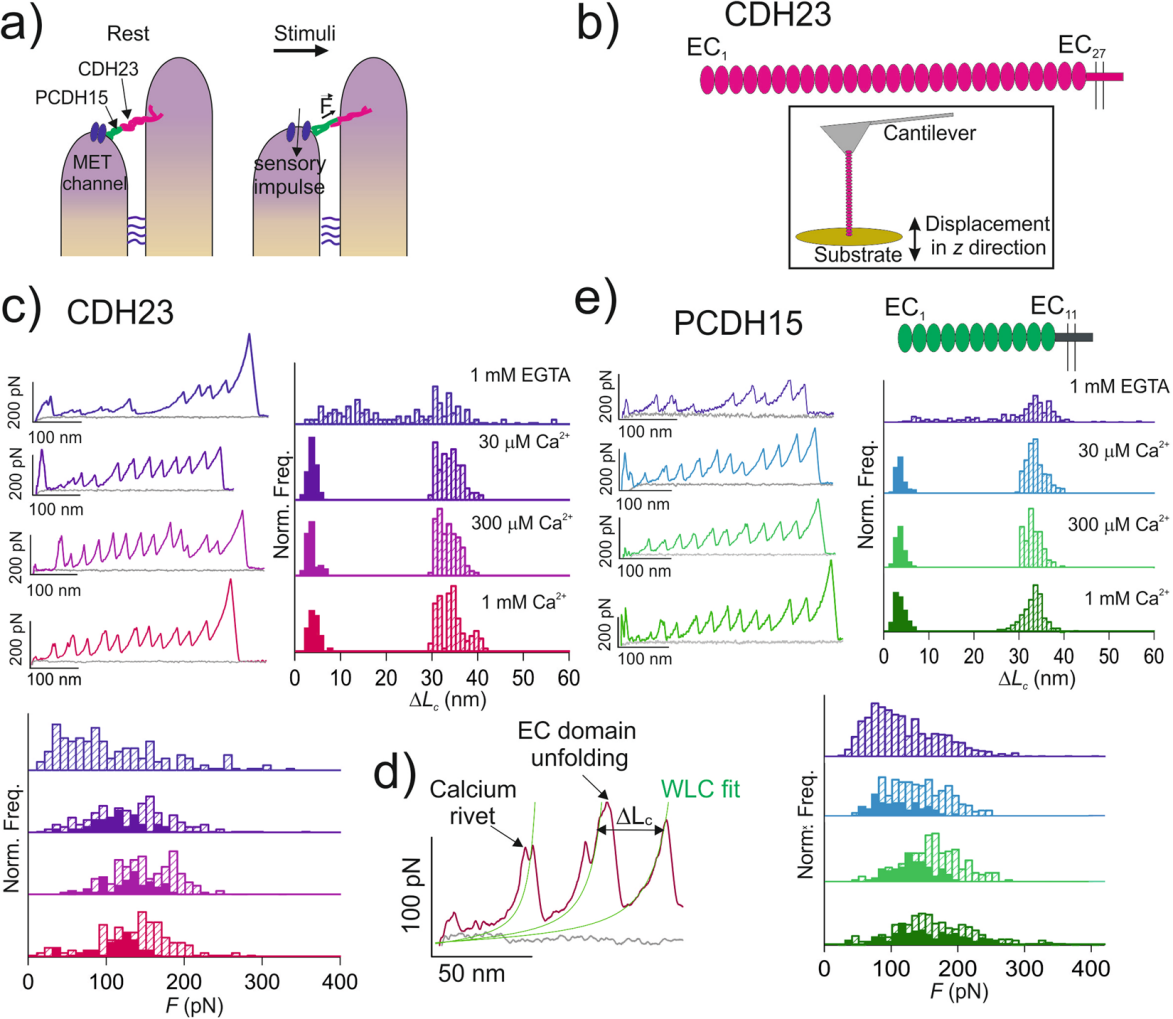
Nanomechanics of the full ectodomain from tip-link cadherins. (**a**) Schematic cartoon of the connections between sterocilia from the hair cells of the inner ear. Tip links are formed by a *trans* interaction of a single *cis* dimer of CDH23 (magenta) and a *cis* dimer of PCDH15 (green). The location of the MET channel is indicated. Lateral connections (indicated with blue horizontal lines) between sterocilia are schematically drawn. (**b**) Typical experimental setup in AFM-SMFS. (**c**) Nanomechanics of full-length CDH23 in different Ca^2+^ conditions. Although we were able to stretch almost 1 μm in *z* direction, no more than 15 peaks were obtained in single-molecule recordings. (**d)** Detailed view of the proximal region of a force-extension recording at 1 mM Ca^2+^ showing calcium rivets^17^ and EC domain unfolding peaks, which are delimited by the WLC fit (in green). (**e**) Nanomechanics of full-length PCDH15 in the mentioned Ca^2+^ conditions. The calcium rivets are marked in the histograms with filled bars (**c,e**). In (**c**) and (**e**) a high number of non-monomeric recordings were obtained, which were discarded following the criteria explained in Methods (Fig. S6c,d). Data is presented from low (top) to high (bottom) Ca^2+^ concentrations (**c,e**).

Hearing and balance perception in vertebrates is considered as one of the most evident mechanotransduction processes^1^. In particular, the hair cells of the inner ear contain a bundle of ascending stereocilia that are deflected by forces produced by undulations that propagate in the endolymph arising from sound waves or head movements^2–7^. These deflections produced by mechanical tension promote the rapid opening of the so-called mechano-electrical transduction (MET) channels and the subsequent depolarization of hair cells^8–10^. This strongly coupled mechano-electrical transduction process requires robust extracellular structures, not only to transmit the tension from the upper to the lower stereocilium leading to the opening of the MET channel, but also to maintain the bundle integrity after continuous mechanical stimulations (Fig. 1a). Among other connectors between the stereocilia, the so-called tip-link, a long intertwined filament formed by a helical dimer of cadherin 23 (CDH23) in the apical end bound head-to-head to a helical dimer of protocadherin 15 (PCDH15) in the basal end, is particularly relevant for the proper gating process and coupling of the MET response^2,4–7^.

Both CDH23 and PCDH15 belong to the Cadherin Superfamily, a large Ca^2+^-binding protein family responsible for cell-cell adhesion^11^. These are single transmembrane proteins with CDH23 containing 27 extracellular cadherin (EC) domains and PCDH15 containing 11 EC domains. It has been shown that Ca^2+^ binding to the tip link is critical for the proper mechanotransduction process in hair cells, since in the absence of this cation the tip-link filament becomes dismantled and signal transduction gets interrupted^8,12^. However, this effect is reversible once extracellular Ca^2+^ levels are restored, then the tip-link filament is rebuilt^13^. Interestingly, several point mutations related with decreased Ca^2+^ binding have also been linked to inherited forms of deafness in humans and rodents, which appear to compromise mechanotransduction^14–16^. We have previously shown the critical role of Ca^2+^ binding in the rigidification and mechanical response of cadherin domains from classical cadherins, and that this binding is also responsible for an additional element of mechanical resistance (extramodular, *i.e*., outside the mechanical clamp of the EC module), termed the “calcium rivet”^17^. Furthermore, we also showed that^17^ the mechanical stability of classical cadherins is significantly reduced at the exceptionally low Ca^2+^ concentration of the endolymph that bathes the tip-link (20–40 μM free Ca^2+^)^18,19^. Still, the tip link is stably formed at 50 μM free Ca^2+^ but if the level of Ca^2+^ is reduced, then the length of the filament increases, probably due to a loss of rigidity or EC domain unfolding^20^. Therefore, tip-link cadherins must show particular Ca^2+^ binding properties compared to classical cadherins, despite the high sequence identity between their Ca^2+^-binding regions and their similar folds^21,22^. However, the intrinsic mechanical properties of this critical mechanotransducer have not been experimentally characterized to date.

Here we report the mechanical properties of the tip-link cadherins and demonstrate that, unlike classical cadherins^17^, these proteins are remarkably mechanostable structures at the low physiologically relevant Ca^2+^ concentration of the endolymph, in agreement with their pivotal role in mechanotransduction. Furthermore, we also show that a single point mutation related to DFNB12 hereditary deafness, which impairs Ca^2+^ coordination^22^, affects the nanomechanics of the domains, but only at physiological Ca^2+^ levels. Taken together, our data provide a necessary molecular insight into this important mechanotransduction system, setting the limits of its proper mechanical response.

## Results

### Nanomechanics of tip-link cadherins

Since tip-link cadherins are crucial components of the mechanotransduction machinery of sound stimuli^1^, their mechanical stability is a critical parameter that will determine their proper function. Moreover, because of their long, multimodular structure and *in vivo* geometry^4^, tip-link cadherins are ideal candidates for single-molecule force spectroscopy (SMFS)^23^. Interestingly, as previously demonstrated for classical cadherins^17^, we can characterize the role of Ca^2+^ coordination modulating the robustness of tip-link cadherins, which is a critical parameter based on the already known effects of Ca^2+^ binding in tip-link formation and rigidity^8,12,22^.

**Figure 2:**
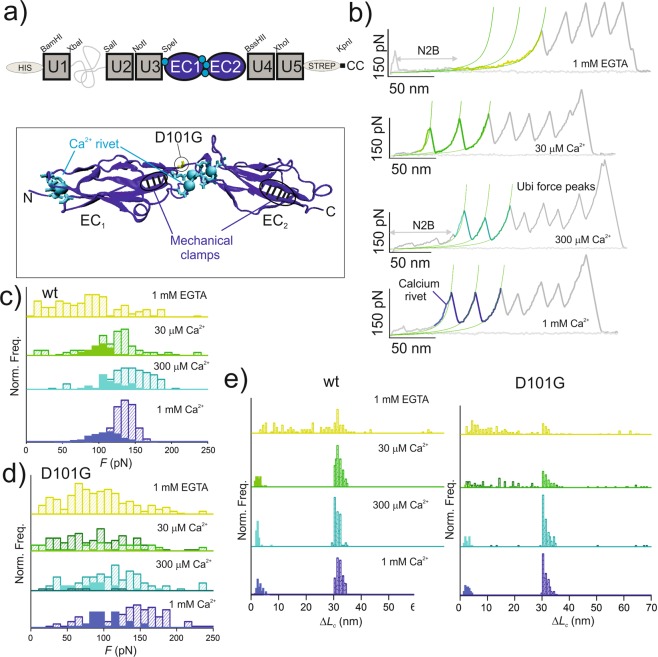
DFNB12 hereditary deafness-associated D101G mutation shows mechanical differences. **(a**) Schematic representation of the pFS + EC_1–2_ construction. The fusion protein pFS-1 was explained in detail elsewhere^25^. The inset at the bottom shows the atomic structure of CDH23 EC_1–2D101G_ domains^22^, showing the coordinated Ca^2+^ ions as cyan spheres, and the residues involved in coordination in bonds representation. Also of note is the characteristic N-terminal Ca^2+^ ion present in tip-link cadherins. The two mechanical determinants showed by these proteins (the mechanical clamps from the EC domains and the calcium rivet^17^) are indicated. The atomic structures shown in this Figure were depicted using VMD 1.8.6^41^. (**b**) WT CDH23 EC_1–2_ domains show canalized unfolding in the low Ca^2+^ concentration present in the endolymph. Several representative force-extension recordings are shown, with the ubiquitin (U in **a**) peaks in grey, the N2B spacer in light grey (belonging to the N2B spring from human cardiac titin, which acts as a spacer from the noisy proximal region of the recordings^25^), and the EC peaks in the corresponding color. The fitting of the WLC model^37^ is shown in green, enabling the observation of the unfolding decanalization of the EC domains in EGTA (top recording), as well as the clear deviation of the calcium rivet from the WLC fitting to the main unfolding peaks (in lighter colors in the spectra). (**c**) *F* histograms of WT EC_1–2_. The *F* values for the rupture of the calcium rivets are shown in solid bars in the histograms. (**d**) *F* histograms of EC_1–2D101G_. In EGTA the values are comparable to those of decanalized EC domains at 30 μM Ca^2+^. (**e**) Unlike the WT, EC_1–2D101G_ showed a high unfolding decanalization in a physiological Ca^2+^ concentration (~30 μM Ca^2+^). For simplicity, only data coming from EC domains are plotted in the histograms.

We have produced full-length murine CDH23 and PCDH15 ectodomains in mammalian cells and analysed their mechanical stability at the single molecule level (Fig. 1). Upon mechanical stretching (Fig. 1b), the full-length CDH23 ectodomain showed the characteristic saw-tooth pattern typical of multi-domain protein unfolding, where each individual force peak represents the unravelling of an individual domain (Fig. 1c). The magnitude of the peak results from the rupture of the main resistance barrier of the domain to mechanical unfolding (the so-called mechanical clamp)^23^, which in cadherin EC domains corresponds to a patch of hydrogen bonds between two parallel *β*-strands that secure the domain^17^ (Fig. 2a). In addition, several force peaks of the recordings are preceded by smaller amplitude peaks with a short increase in contour length (*ΔL*_c_; Fig. 1d) that originates from the rupture of the Ca^2+^ coordination complexes^17^ and disappear at sub-μM Ca^2+^ concentrations (1 mM EGTA, Supplementary Table 1). This additional resistance element to mechanical unfolding known as the calcium rivet, which is also present in classical cadherins upon Ca^2+^ coordination, ensures that the EC domain remains folded when subjected to low range forces^17^. Interestingly, the calcium rivets may break collectively at low loading forces, meaning that several calcium rivets may appear before the first domain unfolding peaks in the recording. After an EC unfolding event and a short relaxation time until the next unfolding event (0.1 s due to the feedback latency of the AFM piezoelectric device), those rivets ruptured at low forces, which are located between EC domains that remain folded, could reassemble giving rise to additional rivets preceding the following force peaks in the recording (Fig. 1d). Therefore, the more Ca^2+^ coordination complexes the protein is able to form, the more evident (with larger *ΔL*_c_) and abundant the calcium rivets will appear in the force recordings. Indeed, the calcium rivets observed for CDH23 (with 27 EC domains, Fig. 1b–d) are more obvious than those shown by classical cadherins (consisting of just 5 EC domains^17^). However, since the rupture of Ca^2+^ coordination networks is a stochastic and cooperative process, not all the EC domain unfolding peaks are preceded by calcium rivets.

Thus, as observed for classical cadherins, CDH23 at saturating Ca^2+^ concentrations showed a “canalized”^17^ mechanical unfolding, consisting of discrete EC domain unfolding events preceded by the rupture of calcium rivets (Fig. 1c,d). However, although the mechanical stability of CDH23 was comparable to that of classical cadherins at saturating Ca^2+^ conditions (Supplementary Table 1^17^), significant differences in the mechanical behaviour of CDH23 are observed at physiological Ca^2+^ concentrations. Specifically, in contrast to classical cadherins, which show “decanalized” (*i.e*. disorganized) unfolding already at 100 μM Ca^2+^ (Fig. S1a–c^17^), CDH23 presented a canalized unfolding in the very low Ca^2+^ concentrations of the endolymph (≈30 μM)^18,19^, meaning that calcium rivets are present at extraordinarily low Ca^2+^ levels showing comparable mechanostabilities (Fig. 1c,d and Supplementary Table 1^17^).

Similarly, full-length PCDH15 also showed a canalized mechanical behaviour at 30 μM Ca^2+^ (Fig. 1e, Supplementary Table 2). Therefore, although there is a wide range of Ca^2+^ affinity values across their whole ectodomains^22,24^, tip-link cadherins contain novel high affinity Ca^2+^ binding sites (Fig. S1d) that enable them to remain resistant to mechanical unfolding even if their Ca^2+^ binding sites are partially saturated at physiological conditions^21,22^. Interestingly, in the case of PCDH15, which contains 11 EC domains^4^, no more than 10 EC peaks were observed per SMFS recording (Fig. 1e); probably the missing EC was denatured or covalently clamped through a disulphide bond^7^, obscured by the noisy proximal region of the recordings^25^, or showed decanalized unfolding because of lack of Ca^2+^ coordination in that particular region^24^. The first peak of the recording corresponds to the detachment of the cantilever tip from the substrate or, alternatively, from the rupture of unspecific interactions from the tip. It is common that the proximal region of the recordings gets obscured by these noisy, non-specific interactions when the protein is detached from the tip or the substrate. The final peak(s) of the recordings corresponds to the detachment of the protein from either the tip or the substrate, which marks the end of the single-molecule experiment^23^.

### A deafness-associated mutation shows a mechanical phenotype

Since tip-link cadherins showed differential Ca^2+^ modulation of their mechanical properties, we aimed to analyze specific domains in different Ca^2+^ concentrations in order to characterize the possible mechanical effect of deafness-related mutations (Figs 2 and S2^15,26,27^). To fully characterize the nanomechanics of the EC modules, we used the pFS-1 polyprotein strategy as a single-molecule marker (Fig. 2a)^25^. We analyzed many individual EC modules as recombinant proteins (Fig. S2) and, although all the individual EC constructs seemed properly folded (in the different Ca^2+^ concentrations tested) in a conformation rich in *β*-structure as measured by CD spectroscopy (Figs S3 and S4), we found only a single construct that enabled a full mechanical characterization: pFS + EC^CDH23^_1–2_. Using this construct, we additionally characterized the D101G deafness-related mutation (Fig. 2), which is known to affect this CDH23 region’s conformation and dynamics, as a consequence of a reduced Ca^2+^ binding^22^. Indeed, the mutation shows a clear mechanical phenotype, but only at low physiological Ca^2+^ concentrations (Supplementary Table 3).

Specifically, both EC_1–2_ WT and its D101G variant showed mechanical stabilities comparable to those of the full-length CDH23 at 1.0 mM Ca^2+^ concentration (Figs 1 and 2 and Supplementary Tables 1 and 3). This observation indicates that the flanking pFS-1 domains (Fig. 2a) do not alter the mechanical properties of the EC modules under study, and therefore are not the source of the altered nanomechanics observed for other EC domains shown in this study (Figs S1 and S2). Remarkably, D101G mutation caused a mechanical phenotype on CDH23 EC_1–2_ reducing the mechanostability and de-canalizing the unfolding, which begins to be apparent at 300 μM Ca^2+^ and becomes more evident at 30 μM Ca^2+^ (Fig. 2c–e and Supplementary Table 3). Thus, it seemed critical to analyse its mechanical properties in physiological conditions. At 300 μM Ca^2+^, 88% of EC_1–2D101G_ molecules (36 out of 41) showed canalized unfolding of both EC domains, while at 30 μM Ca^2+^ this percentage decreased dramatically to 31% (14 out of 45 molecules; Supplementary Table 3). Remarkably, no molecules with decanalized unfolding were observed in these conditions for EC_1–2_ WT, indicating that Ca^2+^ binding to the wild type protein strengthens the EC domains against mechanical unfolding at physiological conditions (Fig. 2e). In contrast, D101G reduces Ca^2+^ binding^22^, compromising the mechanical response of this region of CDH23 (Fig. 2d,e). Indeed, since the proportion of the canalized and decanalized unfolding pathways changes when varying the Ca^2+^ concentration, these results strongly suggest that the sensitivity to Ca^2+^ concentration is altered in the deafness mutant. As a confirmation that a decanalized mechanical unfolding is due to impaired Ca^2+^ binding, both EC_1–2_ WT and EC_1–2D101G_ showed comparable properties in the absence of Ca^2+^ (sub-μM Ca^2+^ concentration) (Fig. 2b–e and Supplementary Table 3).

More notably than in full-length ectodomains (Fig. 1c,e), the average force values of unfolding of EC_1–2_ domains lower with decreasing Ca^2+^ concentrations, likely because EC_1–2_ Ca^2+^ affinity remains in the low range of the average affinity of the whole CDH23 ectodomain, as observed for classical cadherins^28^ (Fig. 2d). As mentioned, this effect is more evident for EC_1–2D101G_. At 30 μM Ca^2+^, EC_1–2D101G_ domains show, on average, 103 ± 63 pN of resistance to unfolding, while the calcium rivet breaks at 129 ± 35 pN (Supplementary Table 3). This result is surprising since the calcium rivets are assumed to work as sacrificial mechanical resistance elements that prevent the unfolding of EC domains, breaking before the main force peak of domain unfolding and showing lower forces^17^. However, only 5 out of 36 molecules showed a calcium rivet, which in turn also show the highest forces values within the distribution for EC unfolding at this condition. Therefore, the *F* value indicated for EC domain unfolding in Supplementary Table 3 contains the large contribution of EC domains that do not show calcium rivet and manifest lower mechanical stabilities, which lowers the overall *F* value.

## Discussion

Channel gating in the inner ear is an extremely sensitive mechanotransduction system in which activation has been recorded with only 3 pN of applied tension and relative displacements of 1 nm or deflections of 1°, with <10 μs of time response^29–32^. Therefore, it is of paramount importance to determine the consequences of differential ion binding on its mechanical response to understand this system’s molecular bases and how Ca^+2^ binding defects uncouple the transmission of mechanical tension to channel gating, producing deafness^3^. We have shown that tip-link cadherins present a canalized mechanical unfolding at the low Ca^2+^ concentrations of the endolymph, in contrast to the behavior of classical cadherins, which show decanalized mechanical unfolding at those concentrations^17^ (Fig. S1a–c). These different properties can be explained as resulting from the differences in coordination affinities displayed by the distinctive Ca^2+^ binding sites present in tip-link cadherins^21,22^ (Fig. S1d). In general, it has been proposed that the specific Ca^2+^-binding affinities of the two most distal EC domains in CDH23 are about 2 orders of magnitude higher than those seen in the equivalent domains of classical cadherins^22,28^. Therefore, it follows that tip-link cadherins have developed a tighter regulation of their function by Ca^2+^ binding. Moreover, since they are assumed to function just as force transmitters, Ca^2+^ binding is expected to strongly determine their mechanical properties. Nonetheless, despite the high conservation of Ca^2+^ coordination sites among cadherins (Fig. S1d), there are remarkable differences in binding affinities^22^. Moreover, D101G mutation largely affects the binding affinities in all binding sites between EC_1–2_ domains^22^, which can be explained in terms of altered rigidity of this interdomain region in the mutant^22^, because of the increased entropy of Gly in the unfolded state. Therefore, a partially rigid interdomain linker is necessary for a proper Ca^2+^ coordination, which in turn provides additional rigidity, as if Ca^2+^ coordination stabilizes particular conformations, *via* a conformational selection mechanism^24,33^. In the future, identifying individual EC domains in the SMFS recordings obtained from full-length ectodomains would be particularly relevant for studying the mechanical effects of deafness-related mutations in the context of the whole protein.

**Figure 3:**
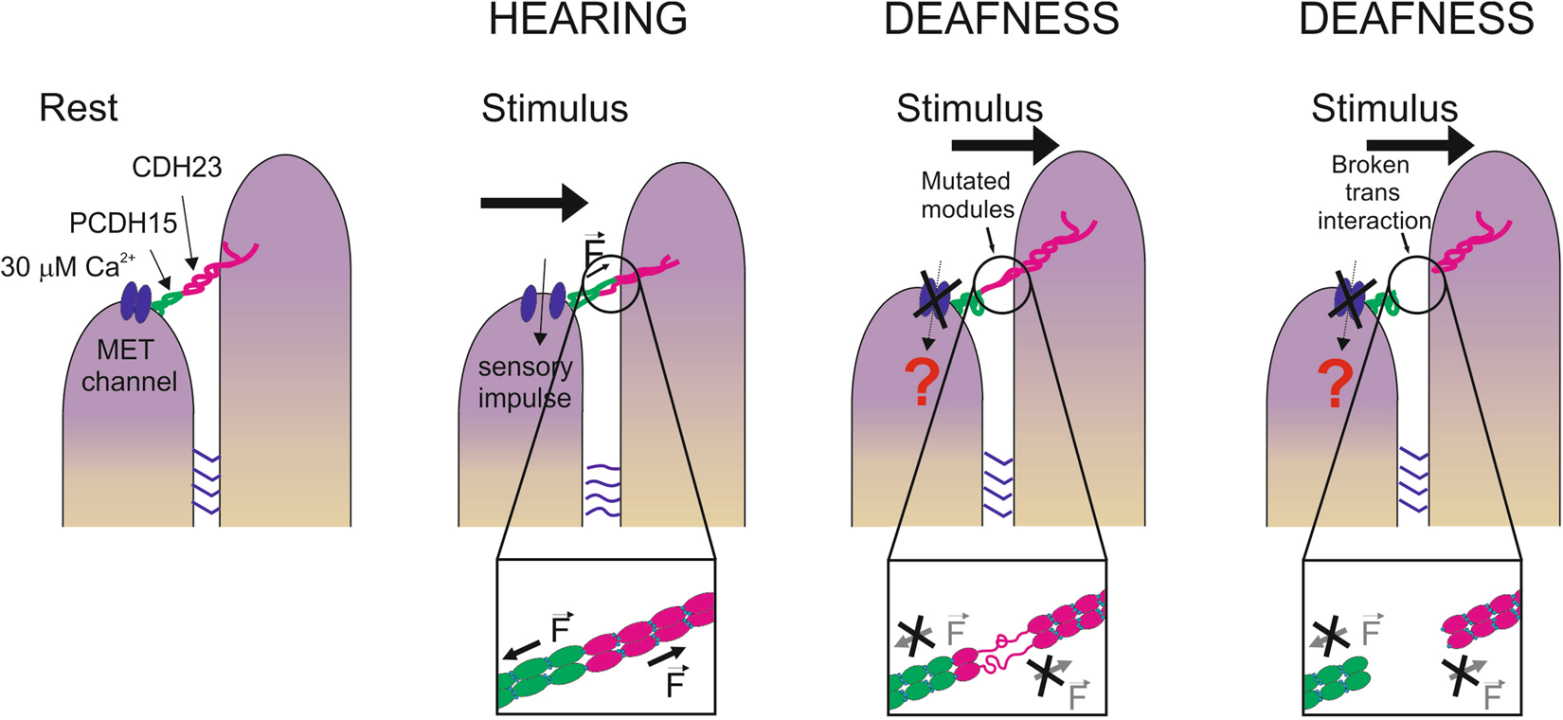
Explanatory model for the mechanical effect of the D101G mutation. After a stimulus, the WT modules will remain folded since they are able to coordinate Ca^2+^ in all the sites and remain canalized. Thus, they will transmit the tension generated by undulations in the endolymph opening the coupled MET channels and generating the hearing response. However, in the case of DFNB12 hereditary deafness-related D101G mutation, the mutated modules will be unable to coordinate Ca^2+^. After a stimulus, they will show decanalized unfolding and will unravel showing no resistance. The tension will not be transmitted to the lower sterocilium, likely uncoupling the mechanism of channel gating and subsequent hearing response, causing deafness. After a continuous stimulation, the reduced mechanical stability of the mutated modules can cause structural defects in the module that could lead to dismantling of the tip link and therefore, the possible interruption of the mechanotransduction circuit.

We have previously proposed the cadherin ectodomain as a “Ca^2+^ switch”, because the mechanical properties dramatically change when the Ca^2+^ concentration is depleted^17^. However, since the main physiological role of cadherin ectodomains is to maintain strong extracellular connections, the EC domains must remain intact at the range of forces supported by these connections. Although the tip link is known to be sensitive to extremely low forces^29^, it is also subjected to continuous mechanical stimuli (Fig. 3). Therefore, for the proper functioning of the tip-link as a force transmitter, tip-link cadherin ectodomains seem to have acquired tighter Ca^2+^ affinity^22^, and have preserved the calcium rivet at the low physiological Ca^2+^ concentration, suggesting that the calcium rivet is an important mechanical component that contributes with additional mechanical resistance to impede the unraveling of the domain upon stretching *in vivo*. D101G shows a reduced number of calcium rivets at physiological Ca^2+^ concentration (Fig. 2) and thus the mutated domains are more exposed to an eventual unfolding event upon stretching, which will uncouple the mechanical transduction system leading to the lack of electrical response to sound stimuli (Fig. 3). Nonetheless, in order to demonstrate the relevance of the calcium rivet and the mechanical phenotype in disease, the mutated domain should unfold before the unbinding of the tip-link. Thus, a single tip-link should resist forces above ∼50 pN^29^, which is the statistical value where many EC D101G domains unfold at the applied loading rate (Fig. 2d).

It must be noted that, statistically, fewer calcium rivet events were observed at the 30 μM physiological Ca^2+^ concentration (particularly for PCDH15, Fig. S5). This is probably because not all the coordination sites were occupied by Ca^2+ ^^22^. Indeed, the mechanical effect observed for the D101G mutant at 30 μM Ca^2+^ (Fig. 2) might be due to incomplete Ca^2+^ coordination. Altogether, our results suggest that when Ca^2+^ is released, WT structures of both CDH23 and PCDH15 fragments are affected, showing differences in secondary structure elements (Figs S3 and S4)^33^ and lower mechanostability (Supplementary Tables 1–3). Interestingly, we were able to observe the reversibility of the “canalization” of domain unfolding upon Ca^2+^ binding (Fig. 1), in line with the reversible Ca^2+^-dependent plasticity of tip-links observed *in vivo*^13^. Furthermore, upon mutation of these proteins, the modules appear destabilized and their mechanical properties get altered showing a lowered mechanostability (Supplementary Table 3).

A decrease in Ca^2+^ concentration causes dismantling of the tip-links^20^. Thus, there is clear correlation between Ca^2+^ coordination, conformational constraints^22^, mechanical stability and tip-link formation, and these properties might determine the proper mechanotransduction in the hair cells^3^. This indicates that slight changes in the mechanical properties of the EC domains can cause long-range effects in the whole tip-link, finally leading to the formation of a less robust complex and the concomitant dysfunction of the mechanotransduction process (Fig. 3)^24^. The effect of other deafness-related mutations in complex formation has been described^3,22,24^. However, our study represents the first experimental evidence of altered mechanical properties of tip-link cadherins at physiological Ca^2+^ concentrations. Because tip-link cadherins play a critical mechanical role in the mechanotransduction process of sound stimuli, the study of their mechanical properties is of paramount importance to understand the molecular mechanisms underlying the transduction of sound and movement stimuli into electrochemical signals. Moreover, the mechanical phenotype of the disease-related mutation studied here is only evident at the low physiological Ca^2+^ concentration, highlighting the importance of characterizing the mechanical properties of tip-link cadherins at their real physiological conditions, rather than using the standard 1.0 mM Ca^2+^ concentration to emulate Ca^2+^ content in extracellular environment.

## Methods

### Protein expression and purification of CDH23 and PCDH15

The His-tagged full murine CDH23 and PCDH15 ectodomains (UniProtKB code Q99PF4 and Q99PJ1, respectively) were expressed in HEK293 cells and purified according to^4^. Cells were cultured in DMEM media supplemented with 10% serum, 1% penicillin/streptomycin and 20 mM HEPES (Gibco). Cell transfection was performed using TurboTransfect (ThermoScientific). Twenty-four hours after transfection, the medium was replaced with F-12 Nutrient Mix (without serum, Sigma). Cells were incubated 48 hours in this medium, when recombinant proteins were overexpressed and secreted to the extracellular media. Supernatants were collected and filtered in Amicon 10-kDa cutoff filters (Millipore) and buffer exchanged to 50 mM TrisHCl/150 mM NaCl/5 mM imidazole [pH 7.5], followed by a Ni^2+^-affinity purification using Histrap HP columns (GE Healthcare). Proteins were concentrated using Amicon filters and buffer exchanged to 50 mM TrisHCl/150 mM NaCl/1 mM CaCl_2_ [pH 7.5]. Protein purity was monitored by SDS-PAGE and Western blotting using anti-Histag antibodies (both proteins contain a C-terminal Histag, see^4^). Proper morphology of the full-length ectodomains was assessed by imaging AFM (see below). Pure proteins were stored at 4 °C at a concentration of ~0.2–0.5 mg/ml.

### Cloning of EC domains

We used the pFS-1 vector, containing ubiquitin repeats as single-molecule markers, to characterize the individual CDH23 and PCDH15 ectodomains (EC) at the single-molecule level^25^. The first two EC domains from CDH23 were analyzed in both the wild type form and containing the D101G mutation (located between both domains)^15^ in pFS-1. An additional construct containing EC_6–8_ from CDH23 was built to analyze the E737V mutation (located between EC_7-8_)^27^ but the pFS + EC_6–8_ fusion protein precluded the proper folding of EC_6–8_ domains (Fig. S2a). In addition, since R139G and G267D mutations are present in PCDH15 EC_1–2_^26^, the first two EC domains from PCDH15 were cloned onto pFS-1, both in WT and mutated form. Since the G267D mutation is located close to EC_2_ C-terminus, similar constructs but containing the first three ECs from PCDH15 were also produced for comparative purposes. None of these EC domains from PCDH15 seemed properly folded when fused to pFS-1, and therefore their nanomechanical properties could not be analyzed (Fig. S2b,c).

The same combinations of domains were individually cloned into the pET28a vector (Novagen) for Circular Dichroism (CD) measurements (Figs S3 and S4). Specifically, EC_1–3_, EC_1–3G267D_ and EC_1–3R139G_ domains from PCDH15 and EC_1–2_, EC_1–2D101G_, EC_6–8_ and EC_6–8E737V_ domains from CDH23 were produced in order to test possible structural effects at different Ca^2+^ concentrations.

### Polyprotein expression and purification

All the polyproteins (pFS + EC_1–2_, pFS + EC_1–2G267D_, pFS + EC_1–3_, pFS + EC_1–3R139G_ and pFS + EC_1–3G267D_ from PCDH15; pFS + EC_1–2_, pFS + EC_1–2D101G_, pFS + EC_6–8_ and pFS + EC_6–8E737V_ from CDH23) as well as the EC domains (EC_1–3_, EC_1–3G267D_ and EC_1–3R139G_ from PCDH15 and EC_1–2_, EC_1–2D101G_, EC_6–8_ and EC_6–8E737V_ from CDH23) were expressed in *E. coli* C41 (DE3)^34^ and BL21 (DE3) strains. Cultures were grown at 37 °C until an OD_600_ of 0.6–0.8 was reached, when the over-expression of the recombinant polyproteins was induced with 1.0 mM IPTG during 4 h at 37 °C, whereas 100 μM IPTG was used to induce the expression of the different EC domain constructs at room temperature for ~16 h. Bacterial pellets were subsequently lysed with 1.0 mg/ml lysozyme and 1% Triton X-100.

Recombinant polyproteins were purified by Ni^2+^ affinity chromatography in Histrap HP (GE Healthcare) columns, using 50 mM TrisHCl/500 mM NaCl/50 mM imidazole [pH 7.5] as binding buffer, adding 500 mM imidazole in the elution buffer. Once eluted, 1.0 mM CaCl_2_ was added to the protein solutions. Fractions with pure protein were concentrated using Amicon 10 K filters (Millipore). A further size-exclusion chromatography was applied in a Hiload 16/60 SD 200 column (GE Healthcare) using 50 mM TrisHCl/150 mM NaCl/1 mM DTT/1 mM CaCl_2_ [pH 7.5] buffer (final experimental buffer). Pure monomeric proteins were concentrated with Amicon 10 K filters (Millipore). Protein purity was monitored by SDS-PAGE and Western blotting as previously described^25^. Recombinant EC domain constructs were purified under denaturing conditions as previously described^22^.

### NTA-Ni ^2+^ functionalization of coverslips for SMFS

We used NTA-Ni^2+^ functionalized coverslips as substrates for SMFS to bind full-length ectodomains from the tip-link cadherins, as well as pFS-1 polyproteins, *via* their C- and N-terminal Histags, respectively. Coverslips were first immersed into a 20 N KOH solution overnight, after which they were transferred to a 2% 3-(mercaptopropyl)triethoxysilane (Sigma-Aldrich), 0.02% acetic acid at 90 °C for 1 h. Afterwards, they were washed in a MilliQ water flow for 1 h and then cured for 15 min in an oven at 120 °C. Next, they were transferred to a 100 mM DTT solution for 15 min and washed under a MilliQ water flow for 1 h. Then, 50 *μ*l of a solution of 20 mg/ml maleimide-C 3-NTA (Dojindo Laboratories) dissolved in 10 mM 3-(N-morpholino)propanesulfonic acid (pH 7) were added and the substrates were incubated for 1/2 h. After a final wash in MilliQ water, a drop of 10 mM NiCl_2_ was added (>50 *μ*l) on the coverslips, incubated for 10 min, and then they were washed with MilliQ water before storage.

### AFM: SMFS and imaging

Approximately 20 μl of the protein sample at ~0.3 mg/ml concentration was adsorbed onto the Atomic Force Microscopy (AFM) substrate (NTA-Ni^2+^-functionalized glass coverslips) via the terminal His-tags and then washed with 50 mM TrisHCl/150 mM NaCl/1 mM CaCl_2_ [pH 7.5]. The buffer composition during SMFS experiments was the same but changing the amount of CaCl_2_ (0.3 mM CaCl_2_, 1 mM EGTA) when required. 30 μM free Ca^2+^ buffer was prepared using diBr_2_-BAPTA as chelating agent^35^. The chemical equilibrium was simulated with the Maxchelator program and the amount of free Ca^2+^ on the experimental buffers was monitored using a Kwik-Tip Ca^2+^ electrode (World Precision Instruments). Recombinant proteins were extensively dialysed against 50 mM TrisHCl/150 mM NaCl [pH 7.5] before further extensive dialysis in the final experimental buffer 50 mM TrisHCl/150 mM NaCl/125 μM CaCl_2_/100 μM diBr_2_-BAPTA [pH 7.5].

All the Single Molecule Force Spectroscopy (SMFS) experiments were performed using the so-called *length-clamp* mode at 0.4 nm/ms constant pulling speed. In this mode, the feedback loop is switched off in the contact region, so that the piezo approaches to the cantilever tip and returns back. The contact portion of the trace was fixed to 0.05% of the total length of the trace. Since the extension in our experiments was performed at 400 nm/s, in general, the total contact time was in the range of 0.1 to 0.5 s, time during which the sample deposited in the substrate can be adsorbed onto the AFM tip *via* non-specific interactions.

The spring constant of each individual atomic force microscope cantilever (MLCT-AUHW, Veeco Probes, with a spring constant of ~40 pN/nm; or BL-RC, Olympus, Tokyo, Japan; with a spring constant of ~30 pN/nm) was calibrated using the equipartition theorem^36^. The length of the protein chain under tension was calculated by fitting the wormlike chain (WLC) model of polypeptide entropic elasticity^37,38^,

$$F(x)=\frac{{k}_{B}T}{p}[\frac{1}{4{(1-x/{L}_{{C}})}^{2}}-\,\frac{1}{4}+\frac{x}{{L}_{C}}]$$

where *F* is the force, *p* is the persistence length, *x* is the end-to-end length, and *L*_c_ is the contour length of the stretched protein. The measured unfolding force of each peak (*F*) in the so-called saw-tooth pattern was pooled and averaged. We also measured the increase in the contour length (*ΔL*_c_) as a measure of the protein length that is force hidden. Values are reported with the standard deviation. At least 4 independent experiments were performed for each condition reported in this study.

In an optimal experiment we can amount to ~10000 trials, 6% of which contains attached protein. The final success rate of analyzable traces is about 2% (194 traces *per* analysis). In the case of CDH23 and PCDH15, this low efficiency could be due to the absence of a fusion protein that favors attachment to the AFM tip by affinity interactions. In addition, CDH23 and PCDH15 full ectodomains were not included inside a single-molecule marker. We did not use here the strategy of flanking domains to avoid compromising the Ca^2+^ coordination efficiency and levels of expression. Therefore, in this case, to be sure that single molecules are being pulled, AFM images and analysis of persistence length had to be performed (Figure S6). In addition, both Δ*L*_*C*_ and the number of unfolded modules were used as single-molecule criteria. Δ*L*_c_ is the criterion used for analysis of those proteins showing “de-canalized” (*i.e*., polymorphic) mechanical properties (Fig. S2b,c)^17^. Regarding the full tip-link ectodomains (Fig. 1), only those recordings showing a saw-tooth pattern with a *ΔL*_c_ close to the expected value for an EC domain (~33–35 nm)^17^ without intercalated peaks, and showing less force peaks than EC domains contained in the protein (27 ECs for CDH23 and 11 for PCDH15) were selected as single-molecule force unfolding recordings. Besides, since these proteins have a high tendency to dimerize (as shown in Fig. S6 and in^4^), the unfolding recordings should additionally show similar persistence length, *p*, across the unfolding curve, indicating that a single polypeptide was being stretched (*p*~0.34 nm)^23^. Indeed, we found several traces showing different *p* values across the unfolding trace, indicating that we often stretched two cadherins in parallel (Fig. S6c,d). Initial CDH23 calcium rivets showed *p* values of 0.47 ± 0.09 nm, while for PCDH15 initial rivets showed 0.49 ± 0.05 nm of persistence length. Once ruptured, *p* values dropped to values ≈ 0.36 nm, corresponding to the unfolding of the polypeptide chain.

Despite the extension of the piezo was set to 1 μm in *z* direction, which allows for full unraveling of tip-link cadherin ectodomains, full-length CDH23 showed an average of 9 ± 1 EC unfolding events *per* force recording, while PCDH15 showed an average of 7 ± 1 unfolded EC domains *per* curve. This indicates that the attachment to the tip was not strong enough to maintain the protein attached for the longer unraveling events. Indeed, we observed total *L*_*c*_ values of 396.0 ± 95.2 nm for CDH23 and 303.3 ± 42.2 nm for PCDH15 (corresponding to 306 and 242 nm of average total *ΔL*_*c*_, respectively). Because the attachment to the tip is random and the protein could be detached at any time, information on preferential or hierarchical mechanical response across the entire ectodomains is not accessible.

AFM images (Fig. S6a,b) were acquired with the protein samples diluted in 50 mM TrisHCl/150 mM NaCl/1 mM CaCl_2_ [pH 7.5]. A drop of the protein solution was deposited on freshly cleaved mica and the *tapping* mode was employed to acquire images in liquid conditions as described in^39^.

### Circular Dichroism spectroscopy

Far-UV CD spectroscopy was performed using a JASCO-J810 spectropolarimeter (JASCO Inc.) equipped with a Peltier temperature control unit and using quartz cuvettes of 1 mm cell-path length. EC domain samples were prepared at 2–5 μM in 15 mM TrisHCl/50 mM NaCl [pH 7.5] changing only the CaCl_2_ concentration (1.0 mM CaCl_2_, 0.3 mM CaCl_2_, 0.03 mM CaCl_2_ and 1.0 mM EGTA) for each specific sample. The corrected spectra were converted into molar ellipticity ([Θ]) using the average molecular masses *per* residue with Spectra Manager software (Jasco Inc.). The CDNN analysis program^40^ was used to monitor the secondary structure content for each construct in each Ca^2+^ concentration.

### Free Ca^2+^ Concentration

Ca^2+^ concentrations in all the experiments shown in this study refer to measured free Ca^2+^ concentrations as described above.

## Supplementary information

Supplementary Information

## Data Availability

Additional relevant data are available from the corresponding authors upon reasonable request.

## References

[1] Gillespie PG, Walker RG (2001). Molecular basis of mechanosensory transduction. Nature.

[2] Ahmed ZM (2006). The tip-link antigen, a protein associated with the transduction complex of sensory hair cells, is protocadherin-15. J Neurosci.

[3] Gillespie PG, Muller U (2009). Mechanotransduction by hair cells: models, molecules, and mechanisms. Cell.

[4] Kazmierczak P (2007). Cadherin 23 and protocadherin 15 interact to form tip-link filaments in sensory hair cells. Nature.

[5] Pickles JO, Comis SD, Osborne MP (1984). Cross-links between stereocilia in the guinea pig organ of Corti, and their possible relation to sensory transduction. Hear Res.

[6] Siemens J (2004). Cadherin 23 is a component of the tip link in hair-cell stereocilia. Nature.

[7] Sotomayor M, Weihofen WA, Gaudet R, Corey DP (2012). Structure of a force-conveying cadherin bond essential for inner-ear mechanotransduction. Nature.

[8] Assad JA, Shepherd GM, Corey DP (1991). Tip-link integrity and mechanical transduction in vertebrate hair cells. Neuron.

[9] Beurg M, Fettiplace R, Nam JH, Ricci AJ (2009). Localization of inner hair cell mechanotransducer channels using high-speed calcium imaging. Nat Neurosci.

[10] Denk W, Holt JR, Shepherd GM, Corey DP (1995). Calcium imaging of single stereocilia in hair cells: localization of transduction channels at both ends of tip links. Neuron.

[11] Halbleib JM, Nelson WJ (2006). Cadherins in development: cell adhesion, sorting, and tissue morphogenesis. Genes Dev.

[12] Vollrath MA, Kwan KY, Corey DP (2007). The micromachinery of mechanotransduction in hair cells. Annu Rev Neurosci.

[13] Zhao Y, Yamoah EN, Gillespie PG (1996). Regeneration of broken tip links and restoration of mechanical transduction in hair cells. Proc Natl Acad Sci USA.

[14] Ahmed ZM (2008). Gene structure and mutant alleles of PCDH15: nonsyndromic deafness DFNB23 and type 1 Usher syndrome. Hum Genet.

[15] Astuto LM (2002). CDH23 mutation and phenotype heterogeneity: a profile of 107 diverse families with Usher syndrome and nonsyndromic deafness. Am J Hum Genet.

[16] Sollner C (2004). Mutations in cadherin 23 affect tip links in zebrafish sensory hair cells. Nature.

[17] Oroz J (2011). Nanomechanics of the cadherin ectodomain: “canalization” by Ca2+ binding results in a new mechanical element. J Biol Chem.

[18] Bosher SK, Warren RL (1978). Very low calcium content of cochlear endolymph, an extracellular fluid. Nature.

[19] Salt AN, Inamura N, Thalmann R, Vora A (1989). Calcium gradients in inner ear endolymph. Am J Otolaryngol.

[20] Furness DN, Katori Y, Nirmal Kumar B, Hackney CM (2008). The dimensions and structural attachments of tip links in mammalian cochlear hair cells and the effects of exposure to different levels of extracellular calcium. Neuroscience.

[21] Elledge HM (2010). Structure of the N terminus of cadherin 23 reveals a new adhesion mechanism for a subset of cadherin superfamily members. Proc Natl Acad Sci USA.

[22] Sotomayor M, Weihofen WA, Gaudet R, Corey DP (2010). Structural determinants of cadherin-23 function in hearing and deafness. Neuron.

[23] Galera-Prat A, Gomez-Sicilia A, Oberhauser AF, Cieplak M, Carrion-Vazquez M (2010). Understanding biology by stretching proteins: recent progress. Curr Opin Struct Biol.

[24] Powers RE, Gaudet R, Sotomayor M (2017). A Partial Calcium-Free Linker Confers Flexibility to Inner-Ear Protocadherin-15. Structure.

[25] Oroz J, Hervas R, Carrion-Vazquez M (2012). Unequivocal single-molecule force spectroscopy of proteins by AFM using pFS vectors. Biophys J.

[26] Ahmed ZM (2003). PCDH15 is expressed in the neurosensory epithelium of the eye and ear and mutant alleles are responsible for both USH1F and DFNB23. Hum Mol Genet.

[27] Schwander M (2009). A mouse model for nonsyndromic deafness (DFNB12) links hearing loss to defects in tip links of mechanosensory hair cells. Proc Natl Acad Sci USA.

[28] Koch AW, Pokutta S, Lustig A, Engel J (1997). Calcium binding and homoassociation of E-cadherin domains. Biochemistry.

[29] Cheung EL, Corey DP (2006). Ca2+ changes the force sensitivity of the hair-cell transduction channel. Biophys J.

[30] Corey DP, Hudspeth AJ (1979). Response latency of vertebrate hair cells. Biophys J.

[31] Corey DP, Hudspeth AJ (1983). Kinetics of the receptor current in bullfrog saccular hair cells. J Neurosci.

[32] Rhode WS, Geisler CD (1967). Model of the displacement between opposing points on the tectorial membrane and reticular lamina. J Acoust Soc Am.

[33] Jin X (2012). Crystal structures of Drosophila N-cadherin ectodomain regions reveal a widely used class of Ca(2)+-free interdomain linkers. Proc Natl Acad Sci USA.

[34] Miroux B, Walker JE (1996). Over-production of proteins in Escherichia coli: mutant hosts that allow synthesis of some membrane proteins and globular proteins at high levels. J Mol Biol.

[35] Bers DM, Patton CW, Nuccitelli R (1994). A practical guide to the preparation of Ca2+ buffers. Methods Cell Biol.

[36] Florin E-L (1995). Sensing specific molecular interactions with the atomic force microscope. Biosensors & Bioelectronics.

[37] Bustamante C, Marko JF, Siggia ED, Smith S (1994). Entropic elasticity of lambda-phage DNA. Science.

[38] Marko JF, Siggia ED (1995). Statistical mechanics of supercoiled DNA. Phys Rev E Stat Phys Plasmas Fluids Relat Interdiscip Topics.

[39] Valbuena A (2007). Quasi-simultaneous imaging/pulling analysis of single polyprotein molecules by atomic force microscopy. Rev Sci Instrum.

[40] Böhm G, Muhr R, Jaenicke R (1992). Quantitative analysis of protein far UV circular dichroism spectra by neural networks. Protein Engineering.

[41] Humphrey W, Dalke A, Schulten K (1996). VMD: Visual Molecular Dynamics. Journal Molecular Graphics.

