# Supplementary material for: Nanomechanics of tip-link cadherins
Source: Sci Rep. 2019 Sep 16;9:13306. doi: 10.1038/s41598-019-49518-x (PMC6746995; doi:10.1038/s41598-019-49518-x)
Supplement: Supplementary file 1 — Supplementary Information [file 41598_2019_49518_MOESM1_ESM.docx]

**Supplementary Material for**

**Nanomechanics of tip-link cadherins**

Javier Oroz, Albert Galera-Prat, Rubén Hervás, Alejandro Valbuena,

Débora Fernández-Bravo & Mariano Carrión-Vázquez


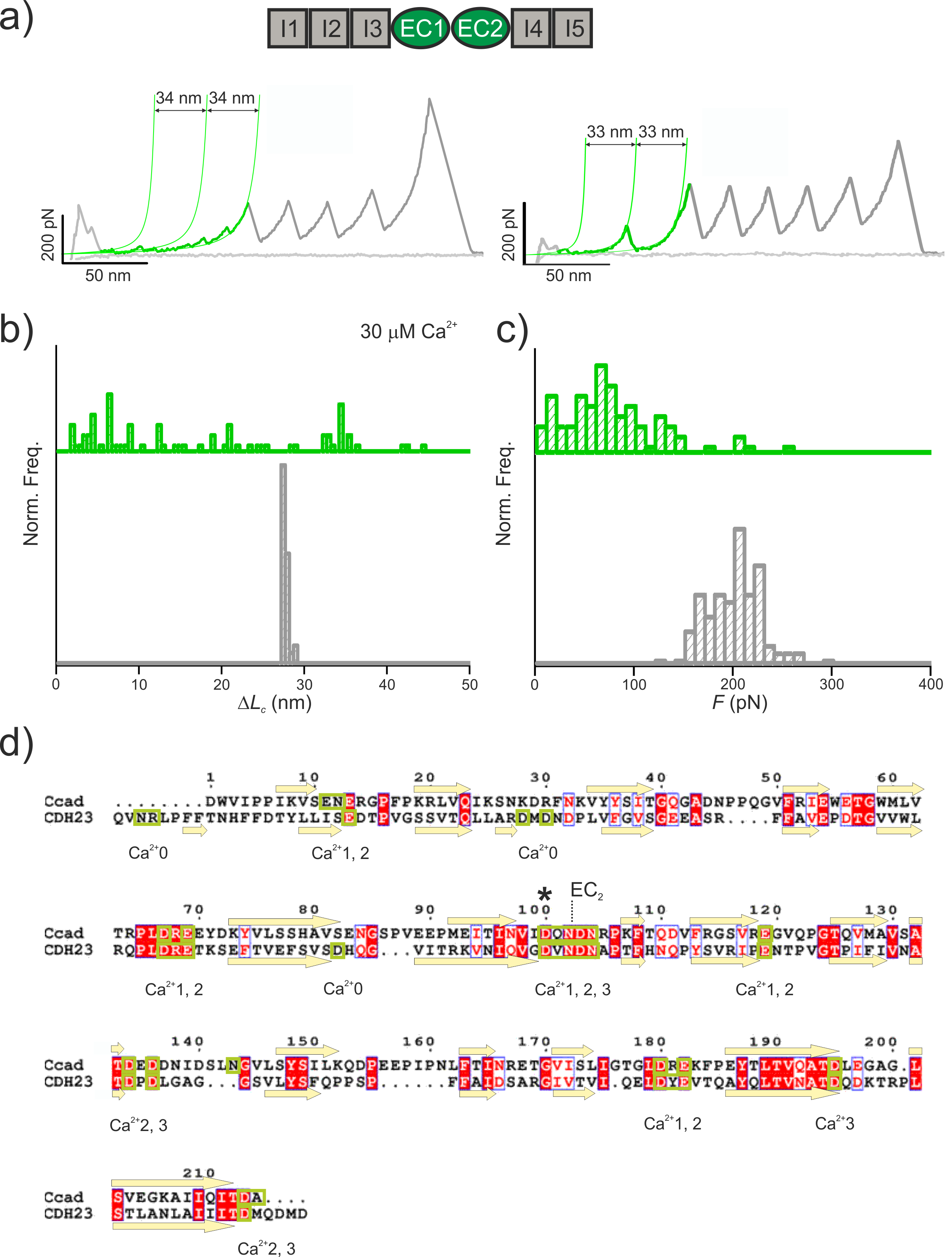


**Figure S1. Control experiments for the unfolding decanalization effects in 30 *μ*M Ca^2+^. a)** Cartoon representation of the heteropolyprotein used for this control experiment; I27 titin repeats are represented in grey boxes indicated with “I” letter and the C-cadherin EC_1-2_ domains are shown in green ovals ^17^. We decided to use this polyprotein since this structure was reported to show decanalized unfolding even at 100 *μ*M Ca^2+^ ^17^. As expected, and contrary to what was shown for tip-link cadherins (**Figs. 1, 2**), these EC domains show decanalized unfolding in 30 *μ*M Ca^2+^. Two representative recordings are shown with the fit of the WLC (green lines) in the data within the region where the unfolding of the EC domains is observed. Some individual EC domains remained canalized (right), but we did not observe any molecule with both domains with canalized unfolding. **b)** Δ*L_c_* histograms for the domains from the heteropolyprotein. The I27 data (grey bars), produced values comparable to previous studies (27.3 ± 0.4 nm; n=96) ^S1^, whereas for the EC domain (green bars) the data show a disperse distribution corresponding to a decanalized unfolding (n= 69). **c)** *F* histograms. Again, the I27 domains produced the expected values in 30 *μ*M Ca^2+^ (200 ± 29 pN) ^S1^, and C-cadherin EC_1-2_ domains manifested a decanalized unfolding (79 ± 53 pN). This result rules out any possible artifactual effect of the buffer composition in 30 *μ*M Ca^2+^ on the mechanical properties, indicating that the effects observed in D101G (**Fig. 2**) specifically originate from the lack of Ca^2+^ coordination compared to WT. **d)** Sequence alignment for EC_1-2_ domains. Ca^2+^-coordinating residues are indicated in green boxes, identical residues in red full boxes and similar residues in blue empty boxes, and the D101G mutation indicated by an asterisk. The *β*-strands are indicated by yellow arrows ^21,22^. Although the global sequence identity is low (25.7%), there is a high conservation among the Ca^2+^-coordinating residues except for the Ca^2+^ coordination at N-terminus (indicated as Ca^2+^ 0), which is characteristic of tip-link cadherins ^21,22^.


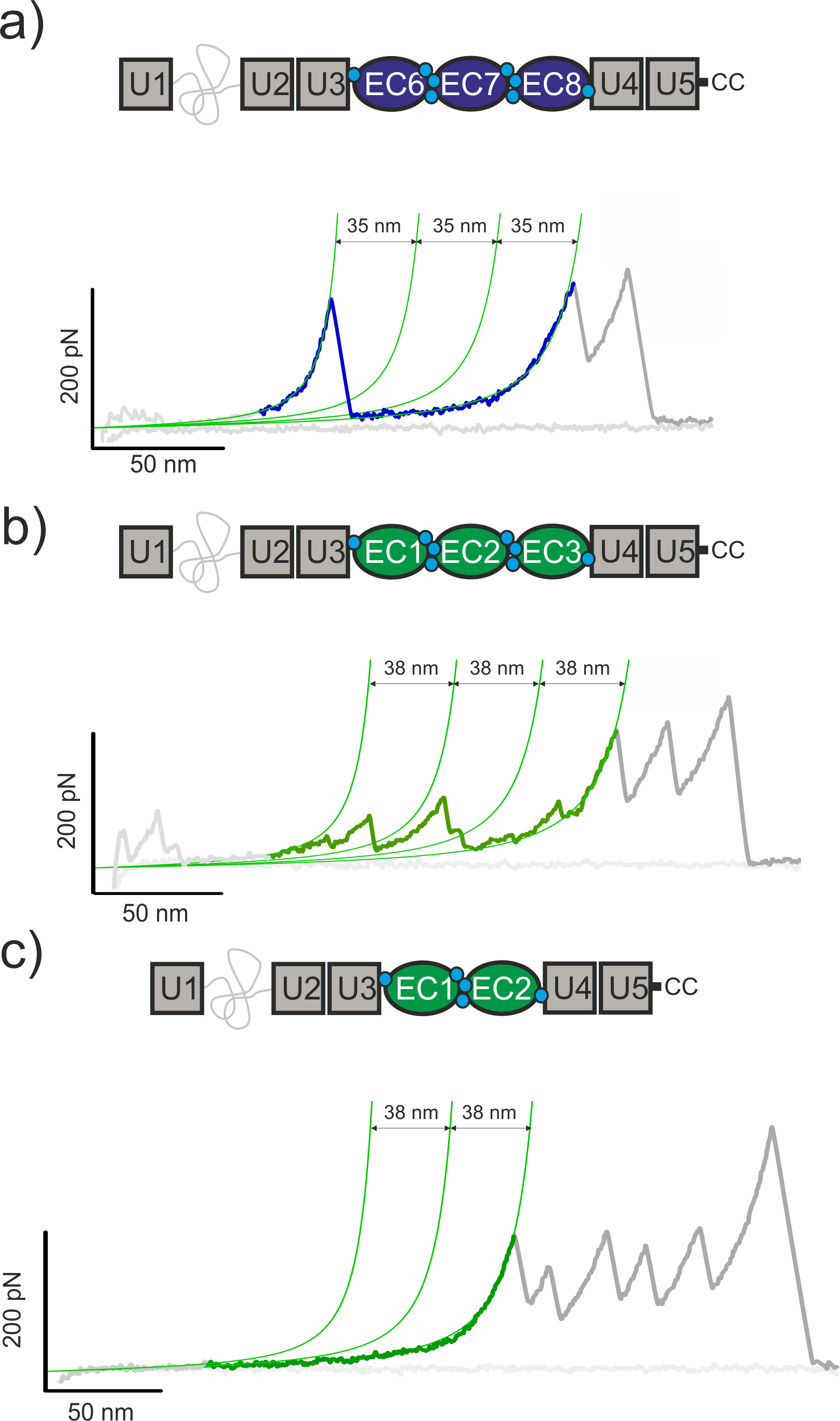


**Figure S2. Representative recordings for CDH23 EC_6-8_ and PCDH15 EC_1-3_ showing the absence of “good” (*i.e*., showing force peaks) recordings.** Although the domains appear to be correctly folded in the different conditions tested by CD (**Figs. S3-S4**), no good SMFS recordings were obtained when they were fused to the heteropolyprotein pFS-1 ^25^, as if these EC domains were denatured or presented decanalized unfolding. Different constructions were used, such as pFS+CDH23 EC_6-8_ (**a**, with the EC domains in blue), pFS+PCDH15 EC_1-3_ (**b**) and pFS+PCDH15 EC_1-2_ (**c**, with the EC domains in green), as well as using another strategy described elsewhere with identical results (**data not shown**) ^S2^. In the recordings, we show the expected Δ*L_c_* observed from the calculated size of the fully unfolded domains as obtained by PSIPRED (36.4 nm for CDH23 EC_6_, 34.8 nm for EC_7_, 36.4 nm for EC_8_; 36.4 nm for PCDH15 EC_1_, 39.2 nm for EC_2_ and 39.2 nm for EC_3_). Since these proteins were not useful for AFM, we were not able to test the possible mechanical effect of other reported mutations related with hereditary deafnesses (E737V in CDH23, and R139G and G267D in PCDH15, ^3,22^). 1.0 mM Ca^2+^ was used in all the spectra shown in this figure.


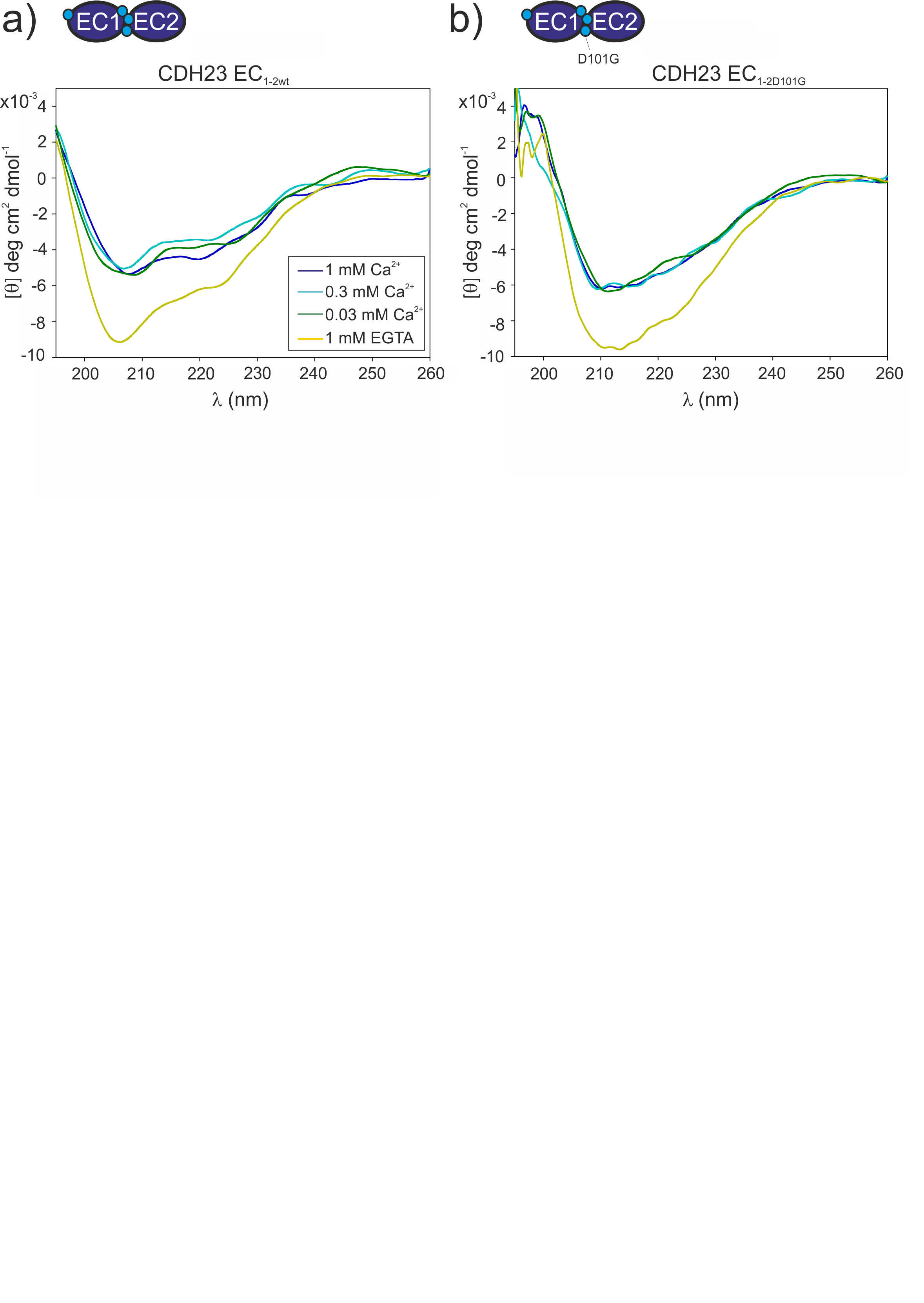
**Figure S3. Secondary structure content of CDH23 EC_1-2_ domains monitored by CD**. **Far-UV CD spectra of WT** CDH23 EC_1-2_ (**a**) and CDH23 EC_1-2D101G_ (**b**) at the specified Ca^2+^ concentrations. The analysis of the CD spectra showed an antiparallel *β* pattern with a [*ϴ*] minimum around 210 nm in all conditions tested, except for the case of EGTA, which produces a significant effect in the secondary structure content in both **WT** CDH23 EC_1-2_ and CDH23 EC_1-2D101G_ constructs, as observed in other cadherin structures ^33^. Upon deconvolution the curves obtained were assigned to various secondary structures using the CDNN analysis program ^40^, showing a major content in antiparallel *β*-structure for all the conditions and proteins, with a decrease in *β*-structure content (while *α*-helical content is increased) for the samples in EGTA. In particular, CDH23 EC_1-2_ showed a decrease in antiparallel *β*-content of 42% in 1.0 mM Ca^2+^ to 36% in 1.0 mM EGTA, whereas CDH23 EC_1-2D101G_ construct changed from 41% antiparallel *β*-content to 34% in the same conditions. These experiments confirm that the observed results for EC_1-2D101G_ in 30 *μ*M Ca^2+^ are not due to structural perturbations in these conditions ^24^. The mentioned Ca^2+^ concentrations in all the experiments shown in this study refer to measured free Ca^2+^ concentrations as detailed in **Methods**. The proteins used for CD measurements consisted only of the EC domains, lacking the rest of the polyprotein elements used for SMFS experiments.


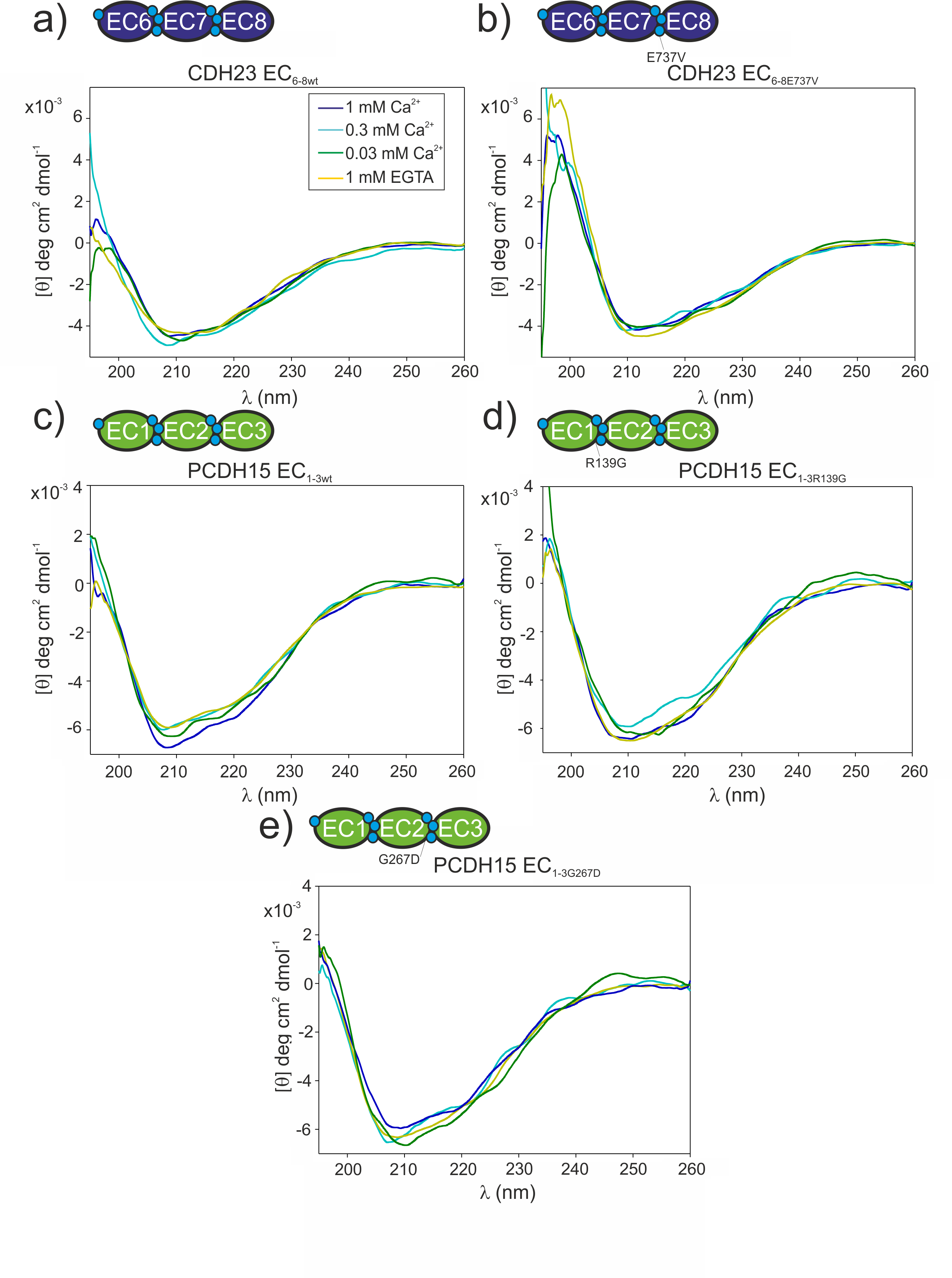
**Figure S4. Secondary structure content of CDH23 EC_6-8_ and PCDH15 EC_1-3_ domains as monitored by CD. Far-UV CD spectra of WT** CDH23 EC_6-8_ (**a**), CDH23 EC_6-8E737V_ (**b**), PCDH15 WT EC_1-3_ (**c**), PCDH15 EC_1-3R139G_ (**d**), PCDH15 EC_1-3G267D_ (**e**). Soluble CDH23 and PCDH15 proteins (in all conditions tested) showed an antiparallel *β*-rich structure as main element of secondary structure, similar to that showed in **Fig. S3**, without significant differences between CDH23 and PCDH15 samples. Thus, although we were unable to obtain robust SMFS data for these proteins and mutants (**Fig. S2**), the domains are folded in all the conditions tested. Antiparallel *β*-content for the proteins (in 1.0 mM Ca^2+^): 43% (**a**), 42% (**b**), 43% (**c**), 44% (**d**), 43% (**e**).

**Figure S5. Ratio of occurrence of calcium rivet rupture *vs*. EC domain unfolding.** D101G mutation significantly reduces the number of observable calcium rivets at physiological Ca^2+^ concentrations. Ca^2+^ concentrations are indicated in the inset. Error bars represent SEM.


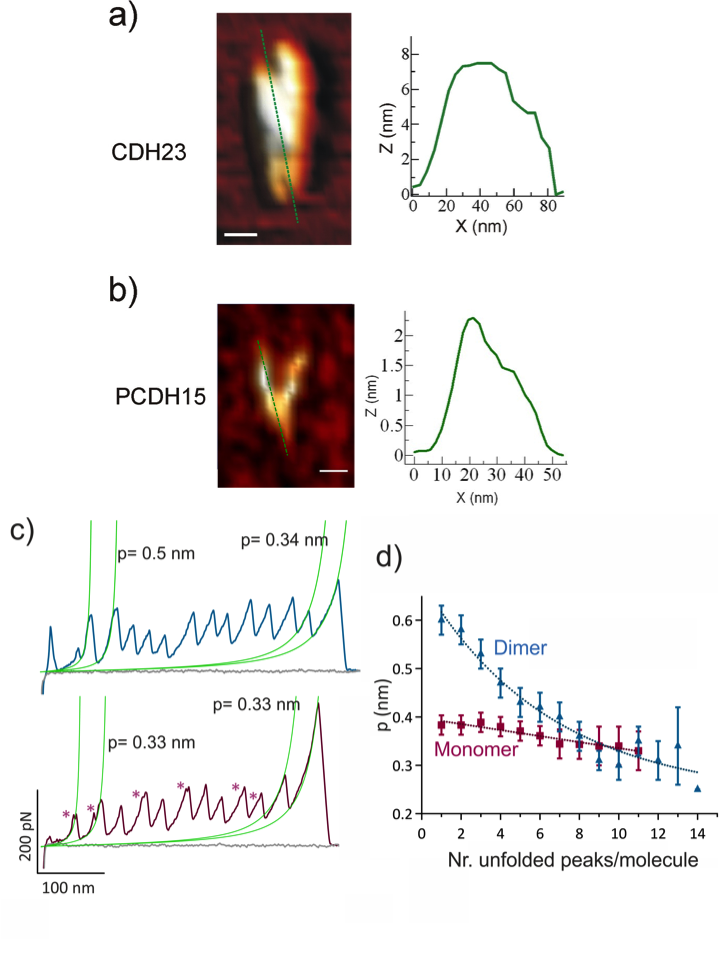


**Figure S6. AFM images of tip-link dimers and associated SMFS patterns. a)** CDH23 dimers. The scale bar corresponds to 30 nm. The right panel shows the cross-section profile (green dashed line on the image). **b)** Separated PCDH15 dimers. Scale bar is 10 nm. The right panel shows the cross-section profile (green dashed line on the image). The height profile shows that PCDH15 (11 modules) dimensions are smaller than CDH23 (27 modules), likely because the protomers are separated. In this case, PCHD15 dimer was disrupted. **c, d)** Mechanical stability of tip-link monomers (purple) and dimers (blue), to explain our observations upon stretching conformations shown in (**a**) and conformations shown in (**b**), which would correspond to single entities. Dimers show larger variation in *p* values and significantly higher unfolding force values. Dimers appear stiffer (larger *p* values) when all modules are still folded. Images were acquired in 1.0 mM Ca^2+^.

**Supplementary Table 1. CDH23 nanomechanics.** At sub-μM Ca^2+^ concentrations, the domain unfolds following a “de-canalized” pattern and no significant Δ*L*_c_ values are obtained. Ca^2+^ rivets are averaged across all unfolding events.

| Ca^2+^ concentration | *F_u_* EC unfolding | Δ*L*_c_ EC unfolding | *F_u_* calcium rivet | Δ*L*_c_ calcium rivet |
| --- | --- | --- | --- | --- |
| 1.0 mM | 138 ± 52 pN (n= 151) | 33.8 ± 3.0 nm  (n= 151) | 110 ± 33 pN (n= 51) | 3.5 ± 1.2 nm  (n= 51) |
| 300 μM | 151 ± 44 pN (n= 219) | 33.1 ± 2.3 nm  (n= 219) | 129 ± 34 pN (n= 81) | 3.2 ± 1.2 nm  (n= 81) |
| 30 μM | 134 ± 59 pN (n= 155) | 33.5 ± 2.6 nm  (n= 155) | 119 ± 45 pN (n= 65) | 3.2 ± 1.0 nm  (n= 65) |
| sub μM (1.0 mM EGTA) | 112 ± 72 pN (n= 152) | - | - | - |

**Supplementary Table 2. PCDH15 nanomechanics.**

| Ca^2+^ concentration | *F_u_* EC unfolding | Δ*L*_c_ EC unfolding | *F_u_* calcium rivet | Δ*L*_c_ calcium rivet |
| --- | --- | --- | --- | --- |
| 1.0 mM | 165 ± 65 pN (n= 359) | 32.7 ± 2.7 nm  (n= 359) | 159 ± 65 pN (n= 197) | 3.2 ± 1.2 nm  (n= 197) |
| 300 μM | 165 ± 47 pN (n= 127) | 32.9 ± 2.1 nm  (n= 127) | 128 ± 33 pN (n= 64) | 3.1 ± 1.0 nm  (n= 64) |
| 30 μM | 142 ± 49 pN (n= 378) | 33.3 ± 2.2 nm  (n= 378) | 109 ± 37 pN (n= 130) | 3.1 ± 1.1 nm  (n= 130) |
| sub μM (1.0 mM EGTA) | 127 ± 67 pN (n= 922) | - | - | - |

**Supplementary Table 3. Nanomechanics of D101G mutant.** For EC_1-2D101G_ in 30 μM Ca^2+^, only 14 out of 45 molecules showed canalized unfolding. The values of the canalized molecules are shown.

| Ca^2+^ concentration | *F_u_* EC_1-2 WT_ unfolding | *F_u_* EC_1-2 D101G_ unfolding | Δ*L*_c_ EC_1-2 WT_  unfolding | Δ*L*_c_ EC_1-2 D101G_  unfolding | *F_u_* calcium rivet EC_1-2 WT_ | *F_u_* calcium rivet EC_1-2 D101G_ | Δ*L*_c_ calcium rivet EC_1-2 WT_ | Δ*L*_c_ calcium rivet EC_1-2 D101G_ |
| --- | --- | --- | --- | --- | --- | --- | --- | --- |
| 1.0 mM | 133 ± 18 pN  (n= 310) | 136 ± 49 pN  (n= 92) | 31.9 ± 1.2 nm (n= 310) | 31.2 ± 1.2 nm (n= 92) | 107 ± 21 pN  (n= 105) | 102 ± 24 pN  (n= 32) | 2.8 ± 1.1 nm (n= 105) | 2.3 ± 0.9 nm (n= 32) |
| 300 μM | 139 ± 32 pN  (n= 76) | 110 ± 50 pN  (n= 73) | 31.0 ± 1.1 nm (n= 76) | 31.2 ± 1.4 nm (n= 73) | 116 ± 21 pN  (n= 24) | 86 ± 37 pN  (n= 18) | 2.2 ± 1.1 nm (n= 24) | 2.3 ± 0.9 nm (n= 18) |
| 30 μM | 121 ± 52 pN  (n= 82) | 103 ± 63 pN  (n= 36) | 31.6 ± 1.2 nm (n= 82) | 31.3 ± 1.3 nm (n= 36) | 105 ± 26 pN  (n= 25) | 129 ± 35 pN  (n= 5) | 2.3 ± 1.0 nm (n= 25) | 2.8 ± 1.4 nm (n= 5) |
| sub μM (1.0 mM EGTA) | 89 ± 51 pN (n= 106) | 94 ± 48 pN (n= 148) | - | - | - | - | - | - |

**Supplementary references**

S1 Oroz, J. *et al.* The Y9P variant of the I27 module: structural determinants of its revisited nanomechanics. *Structure* **24,** 606-616 (2016).

S2 Hervas, R. *et al.* Common features at the start of the neurodegeneration cascade. *PLoS Biol* **10**, e1001335 (2012).
